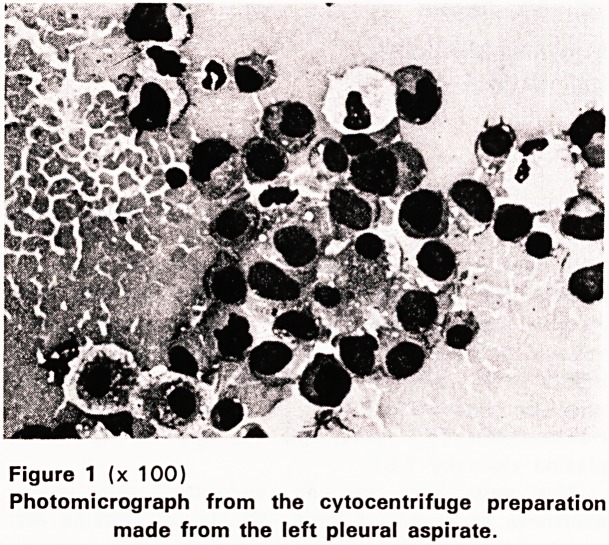# Plasma Cell Sarcoma Complicating a Case of Multiple Myeloma

**Published:** 1976

**Authors:** Saud A. Sejeny, C. M. Asplin

**Affiliations:** Departments of Haematology and Medicine, Southmead Hospital, Bristol; Departments of Haematology and Medicine, Southmead Hospital, Bristol


### Plasma Cell Sarcoma Complicating a Case of Multiple Myeloma

**Saud A. Sejeny and C. M. Asplin**
Departments of Haematology and Medicine,
Southmead Hospital, Bristol

### INTRODUCTION

Several authors have drawn attention to malignant transformation as a terminal feature of multiple myelomatosis (Editorial, *B.M.J.*, 1971). Both acute leukaemia and plasma cell sarcoma have been described, and it has been suggested that the malignant change may be related to treatment.

We describe a further case of multiple myelomatosis terminating in a plasma cell sarcoma in which the striking features were massive involvement of the pleura, the cytological examination of pleural aspirate to make the diagnosis and the rapid onset of the second malignancy.

### Case Report

A 52-year-old plant operator presented in September 1972 with a two month history of progressive shortness of breath and palpitations. He had been previously well apart from troublesome nose bleeds over the past four years. Examination revealed pallor, bleeding from Little's area of the nostrils and scattered retinal haemorrhages.

### Investigations

Haemoglobin 8.0 g/dl (normal indices), normal WBC, plasma viscosity 3.64 cp (normal 1.50-1.72 cp), total protein 138 g/l (albumen 18 g/l discrete band in gamma region). IgG 98 g/l (normal 8.4-17.0), IgA less than 0.27 (1.4-4.2), IgM less than 0.34 (0.5-1.9). The sternal marrow was infiltrated with large numbers of grossly abnormal plasma cells. Chest X-ray and skeletal survey normal. Creatinine clearance 100 mls. per minute. No Bence-Jones protein detected.

Treatment was started with plasmapheresis, transfusion, melphalan and prednisolone. Nose bleeds were eventually controlled after 30 units of plasmapheresis and 10 units of packed red cells.

Following this he remained well for fourteen months with intermittent melphalan (10 mg.) and prednisolone (40 mg.) for 7 days every six weeks.

In February 1974 he was readmitted with a tender, swollen left calf. Chest clear, chest X-ray and ECG normal. He was anticoagulated for a presumed deep venous thrombosis. He attended for follow-up one month later, and was perfectly well with normal physical examination and a normal chest X-ray. Haemoglobin 12.1 g/dl, IgG 16, IgA 1.3, IgM 1.0, plasma viscosity 1.52.

Five days later he was readmitted with severe shortness of breath, a large left pleural effusion and hepatosplenomegaly. Skeletal survey was normal, haemoglobin 8.0 g/dl, WBC 8.9 x $10^9$/1 (1.07 primitive mononuclears), platelets 20 x $10^9$/1, plasma viscosity 3.41 cp. IgG 100, IgA less than 0.27, IgM less than 0.34. Creatinine clearance 100 ml. per minute. Bence Jones proteinuria not detected.

The chest was aspirated, yielding 500 ml. clear fluid. On cytocentrifugation, large numbers of abnormal plasma cells were seen. The bone marrow was replaced by large numbers of primitive cells. Warfarin was stopped and vitamin K, blood and platelet transfusions given. Chemotherapy with the TRAP regime started. (Thioguanine 100 mg. and prednisolone 30 mg. per square metre orally for five days, daunorubicin 40 mg. per square metre intravenously on the first day and cytosine arabinoside 100 mg. per square metre intravenously daily for five days.)

Repeated chest aspirations were required and a total of five litres of fluid were removed. He died before the first course of chemotherapy was completed.

### Comment

The striking features in this case were the findings in the bone marrow and pleural aspirate, when the patient suddenly presented with severe shortness of breath in April 1974. The marrow was heavily infiltrated with bizarre mononuclear cells which constituted 63% of the total nucleated cells. These cells were large and appeared as plasmacytoid cells showing abnormal mitoses with active and abundant cytoplasm with occasional vacuolation. Erythropoiesis and granulopoiesis were markedly depressed. Megakaryocytes were scenty. Cytochemical study revealed pyroninophilic cells which were PAS negative. The reticulin pattern was slightly increased in the histological section of the bone marrow. A cytocentrifuge preparation of the pleural aspirate showed large numbers of strikingly similar cells (Figure 1).

**Figure 1** (x 100)
**Photomicrograph from the cytocentrifuge preparation made from the left pleural aspirate.**

**Post Mortem** (Dr. C. R. Tribe)

The left pleural cavity was completely filled with heavily bloodstained fluid and there were numerous soft, pink, dome-shaped tumour nodules varying in size up to 5 cm. in diameter studded over the visceral and parietal pleura. Similar tumour nodules were present in the anterior mediastinum and the mediastinal lymph nodes were involved.

Histological sections of a pleural tumour show large numbers of plasma cells, varying in size and shape with many multi-nucleated and giant cell forms. The liver, spleen and vertebral bone marrow showed diffuse infiltration with similar cells.

**Discussion**

The termination of multiple myeloma in a widely disseminated myelogenous leukaemia has been reported by several investigators. Nordenson (1966) in a review of 310 cases of multiple myeloma found two cases which developed into acute myelomonocyte leukaemia and a further five cases terminating in acute lymphoblastic leukaemia. Andersen and Vidabaek (1970) reported four out of nineteen cases of myeloma in which myelomonocytic leukaemia developed during the course of treatment with melphalan and/or cyclophosphamide. Kyle and Pierre (1970) reported four cases of myeloma terminating in myeloblastic leukaemia. These authors raised the possibility that cytotoxic drugs might be leukaemogenic. In all these cases they showed at the terminal stage a diffuse leukaemic infiltration of the bone marrow and the peripheral blood, despite return of immunoglobulin levels to normal.

Holt and Robb-Smith (1973) described three patients with multiple myeloma who developed a plasma cell sarcoma during the course of treatment and suggested that the malignant transformation was a result of therapy.

In the case presented here, the sudden development of severe shortness of breath was a result of the massive pleural effusion due to infiltration of the pleura by tumour. The peripheral blood showed 12% of atypical mononuclear cells which closely resembled those found in the bone marrow and pleural fluid.

It should be recognised that a significant number of cases of myelomatosis will terminate in plasma cell sarcoma. In view of the possibility that this change may be related to therapy, it is important to approach with caution the question of long term treatment with cytotoxic agents in patients with symptomless myelomatosis.

**Acknowledgments**

We are grateful to Dr. J. Verrier Jones for permission to publish this case, and to Dr. F. J. W. Lewis and Dr. I. D. Fraser for encouragement and advice.

**REFERENCES**

Andersen, E. and Vidabaek, Aa. (1970). Stem cell leukaemia in myelomatosis. Scandinavian Journal of Haematology, **7**, 201.

Editorial (1971). Leukaemia on myeloma. British Medical Journal, **1**, 568.

Holt, J. M. and Robb-Smith, A. H. T. (1973). Multiple myeloma: Development of plasma cell sarcoma during apparently successful chemotherapy. Journal of Clinical Pathology, **26**, 649.

Kyle, R. A., Pierre, R. V. and Bayrd, E. D. (1970). Multiple myeloma and acute myelomonocytic leukaemia. New England Journal of Medicine, **283**, 1121.

Nordenson, N. G. (1966). Myelomatosis. A clinical review of 310 cases. Acta Medica Scandinavica, 179, Supplementum 445, 178.

## Figures and Tables

**Figure 1 f1:**